# Supplementary material for: Voluntary rewards mediate the evolution of pool punishment for maintaining public goods in large populations
Source: Sci Rep. 2015 Mar 10;5:8917. doi: 10.1038/srep08917 (PMC5390906; doi:10.1038/srep08917)
Supplement: Supplementary Information — Supplementary text and figures [file srep08917-s1.pdf]

Supplementary Information for

Voluntary rewards mediate the evolution of pool punishment for maintaining public goods in large populations

Tatsuya Sasaki[1,*], Satoshi Uchida[2], Xiaojie Chen[3]

[1]Faculty of Mathematics, University of Vienna, 1090 Vienna, Austria

[2]Research Center, RINRI Institute, 101-8385 Tokyo, Japan

[3]School of Mathematical Sciences, University of Electronic Science and Technology of China, 611731 Chengdu, China

*Corresponding to: tatsuya.sasaki@univie.ac.at

This file include:

Supplementary Text, S1

Supplementary Figures, S1-S7

Legends for supporting figures, S1-S7

Text S1 Model variants

Quadratic benefit functions. We can investigate some variants in benefit functions, which in the main text have been linear proportionally to $c_1 r_1$. Here we extensively examine quadratic functions for the provision of the total benefit, as follows: $B(X) = c_1(r_{12}X^2 + r_1X)$, in which X denotes the number of contributors to the public good: $X = n_C + n_P + n_R$. The first term in the right side of equation (1) is replaced with $B(X)/(n - n_N)$. In Supplementary Fig. S7 it turns out that concavity with $r_{12} < 0$ or convexity with $r_{12} > 0$ can lead the center point Q_{RC} on the CDR face to turn, respectively, into an attractor or a repeller (Supplementary Fig. S7).

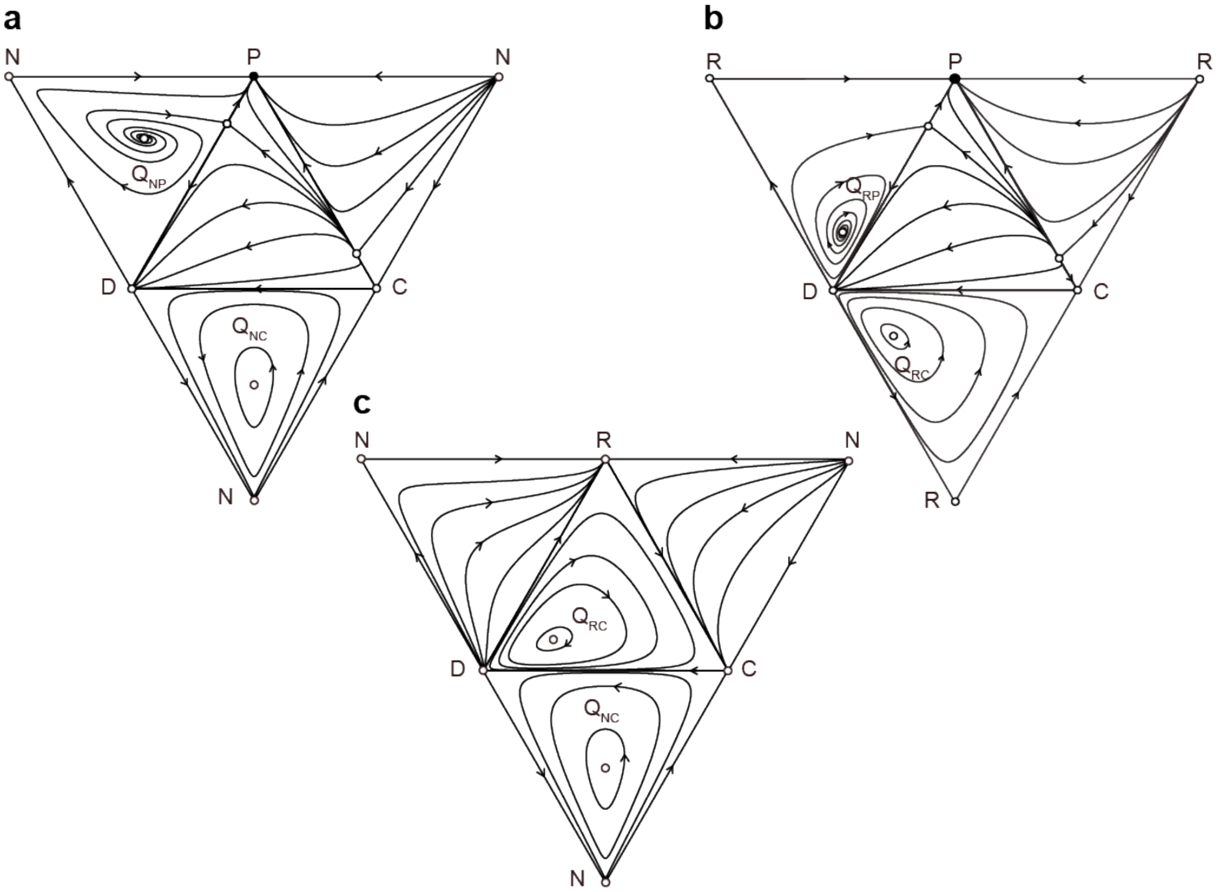

Figure S1 | Replicator dynamics on boundaries. Public good games with (**a**) optional participation and pool punishment, (**b**) compulsory participation and pool reward, or (**c**) optional participation and pool reward. Each simplex component describes a phase portrait associated with the replicator dynamics for three strategies displayed at the corners. *Open* and *filled* circles denote unstable and asymptotically stable equilibria, respectively. The corresponding 3-D phase portrait for four strategies is given in Figs. 1b, 1c, or 3.We note that for panels **a** and **b** the evolutionary dynamics are qualitatively similar on the faces. Indeed, the CDP face is common. And, the replicator dynamics on CDN in panel **a** and CDR in panel **b** lead to cyclical oscillations, on CPN in panel **a** and CPR in panel **b**, to bistability for P and C, and on DPN in panel **a** and DPR in panel **b**, to a repeller on the face and convergence to P. Despite this fact, interestingly, the interior dynamics, demonstrated in Figs. 1b and 1c, are strikingly contrast to each other in the aspect of bistability. Parameter values are: $n = 5$, $c_1 = 1$, $r_1 = 3$, $c_2 = 1$, $r_2 = 2$, $c_3 = 0.1$, $r_3 = 1.6$, $k_{RP} = 2$, $k_{PC} = 1$, and $g = 1$, as in Fig. 1.

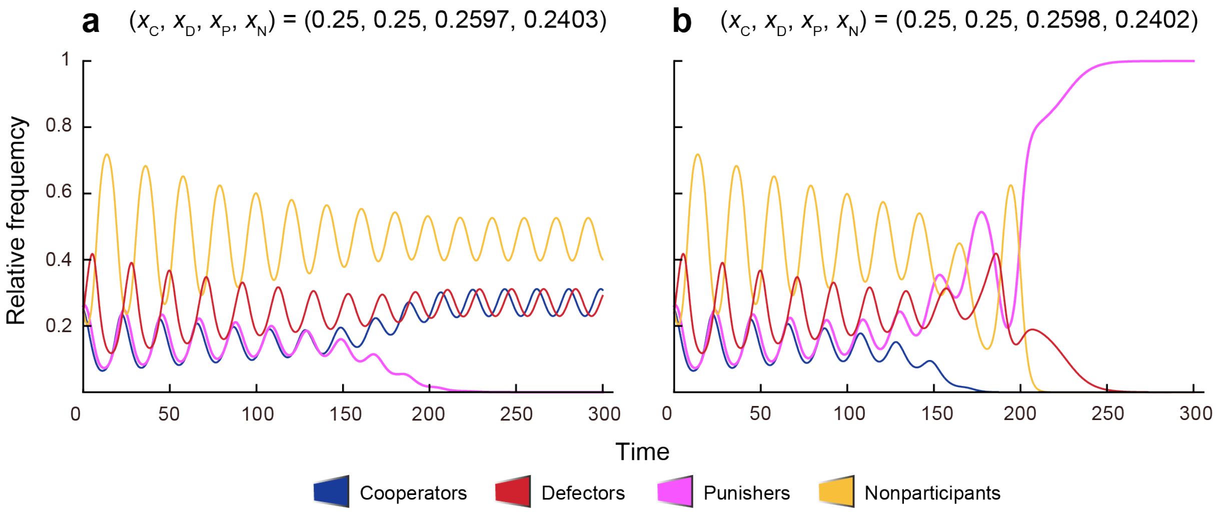

Figure S2 | Sensitive responses to initial conditions in optional public good games with pool punishment. Time series of the frequencies of four strategies, C (*blue*), D (*red*), P (*purple*), and N (*yellow*), corresponding to Fig. 1b. (**a**) The population continues periodically oscillating while the frequency of P gradually decreases and finally vanishes. (**b**) The initial frequencies of P and N are only 0.0001 more are less than those in panel **a**. The population starts with similarly oscillating, then instead of P-players, C-players first vanish, followed by extinction of N- and D-players. This leads to attaining the all-P state (which is stable by second-order punishment). Parameter values are as in Fig. 1. Initial conditions are: (x_C, x_D, x_P, x_N) = (0.25, 0.25, 0.2597, 0.2403) for panel **a** or (0.25, 0.25, 0.2598, 0.2402) for panel **b**. The system has second-order punishment.

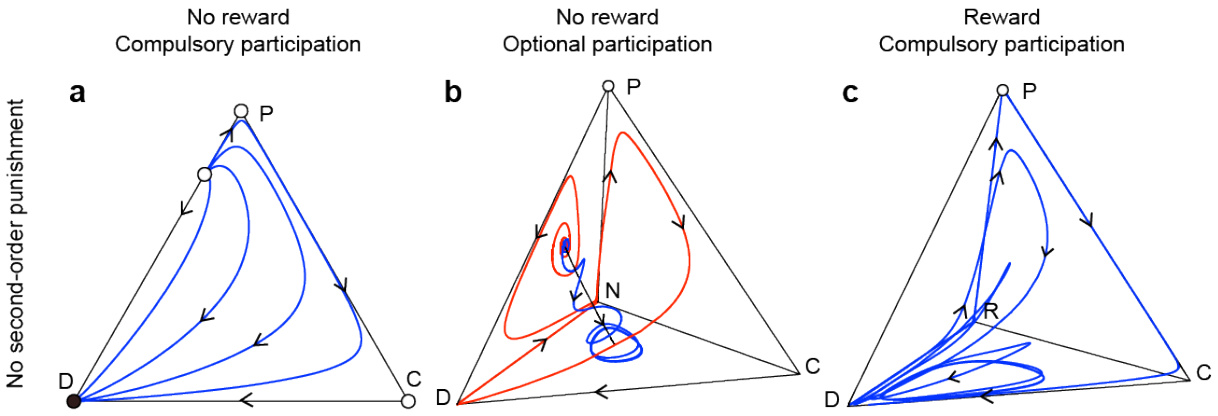

Figure S3 | Evolution of pool punishment without second-order punishment. The P node is no longer stable. (**a**, **b**) With no reward. The population can converge to the D node in compulsory participation in panel **a** or the CDN face in optional participation in panel **b**. (**c**) With reward. The population can converge to the heteroclinic cycle connecting the nodes C, D, R, and P in compulsory participation. Parameter values are: $n = 5$, $c_1 = 1$, $r_1 = 3$, $c_2 = 1$, $r_2 = 2$, $c_3 = 0.1$, $r_3 = 1.6$, $k_{RP} = 2$, $k_{PC} = 1$, and $g = 1$. *Open* and *filled* circles denote unstable and asymptotically stable equilibria, respectively.

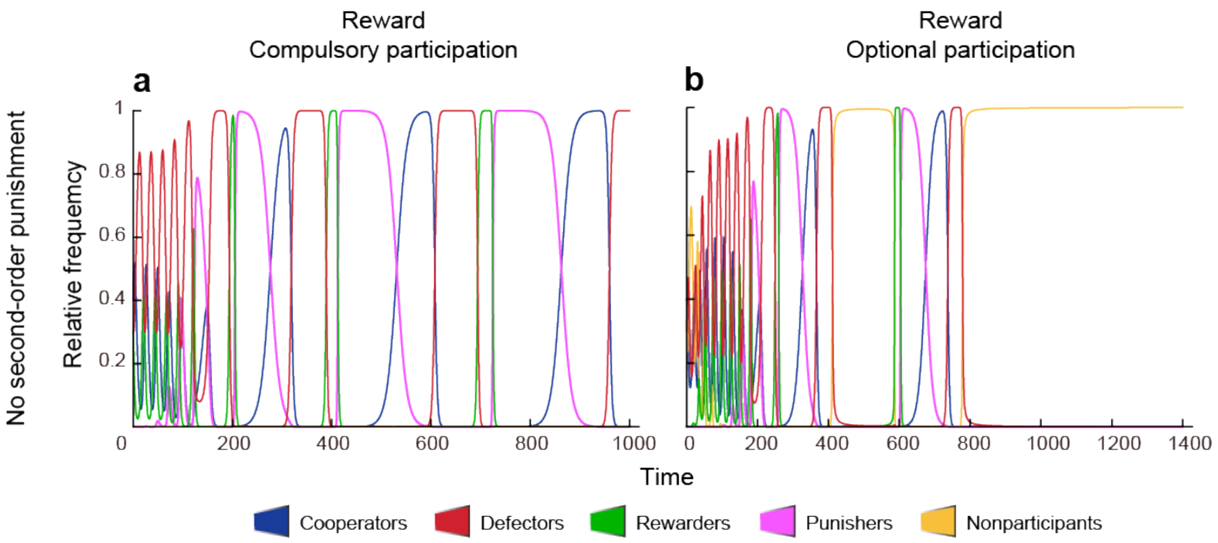

Figure S4 | Cycles without second-order punishment. Time series of the frequencies of five strategies C (*blue*), D (*red*), P (*purple*), R (*green*), and N (*yellow*). The system has no second-order punishment. The homogeneous state of P is no longer stable. Instead the population converges to heteroclinic cycles; in particular with optional participation in panel **a**, the population will stay in the homogeneous state of N for a long time. Parameter values are as in Fig. 1. Initial conditions are: $(x_C, x_D, x_P, x_R, x_N)$ = (0.4, 0.2999, 0.0001, 0.3, 0) for panel **a**, or (0.4, 0.2998, 0.0001, 0.0001, 0.3) for panel **b**.

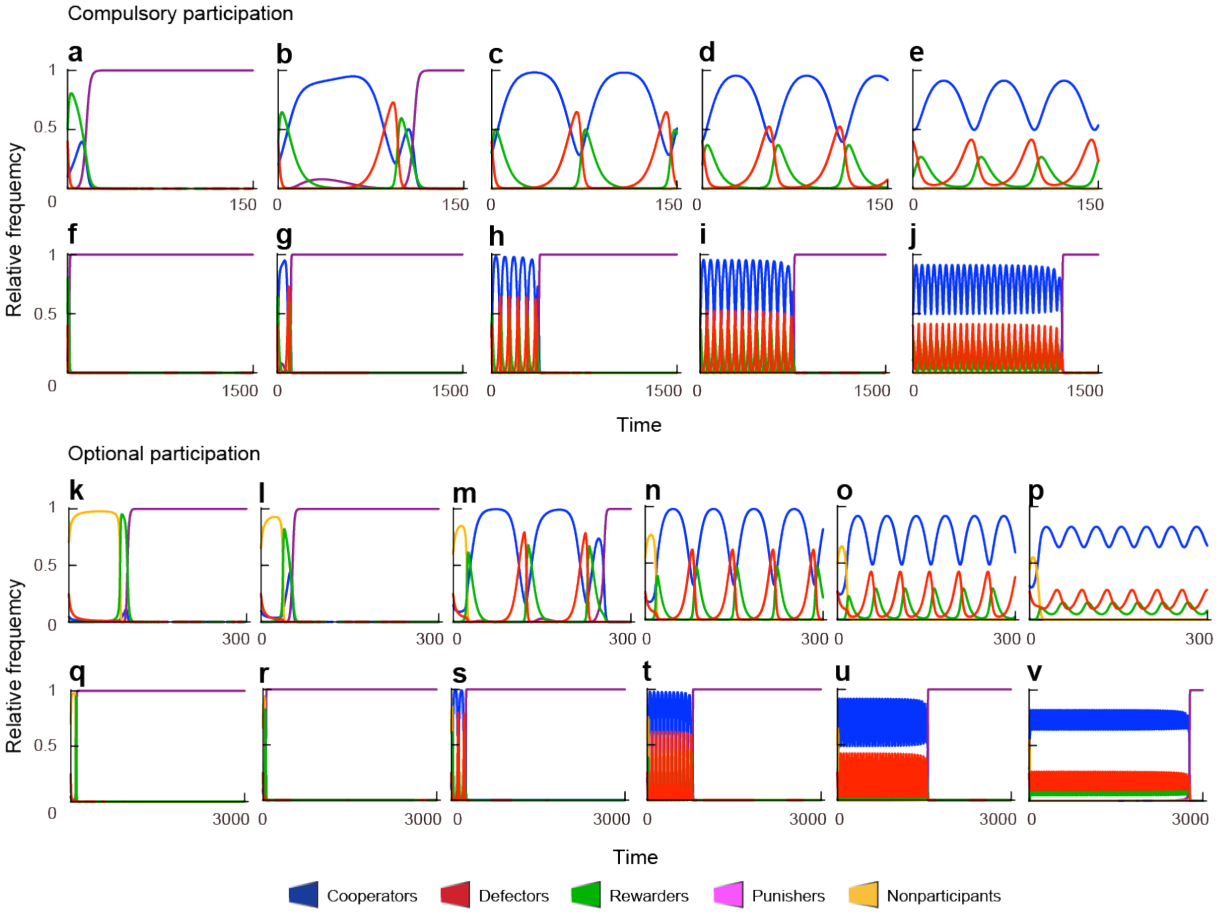

Figure S5 | Effects of initial conditions in compulsory/optional public good games with pool reward and punishment. Time series of the frequencies of (**a-j**) four strategies, C (*blue*), D (*red*), R (*green*), and P (*purple*), (**k-v**) five strategies, C, D, R, P, and N (*yellow*). Parameter values are as in Fig. 1. Initial conditions are: (x_C, x_D, x_P, x_R, x_N) = (0.1, 0.39999, 0.00001, 0.5, 0) for panels **a** and **f**, (0.2, 0.39999, 0.00001, 0.4, 0) for panels **b** and **g**, (0.3, 0.39999, 0.00001, 0.3, 0) for panels **c** and **h**, (0.4, 0.39999, 0.00001, 0.2, 0) for panels **d** and **i**, (0.5, 0.39999, 0.00001, 0.1, 0) for panels **e** and **j**, (0.05, 0.24998, 0.00001, 0.00001, 0.7) for panels **k** and **q**, (0.1, 0.24998, 0.00001, 0.00001, 0.65) for panels **l** and **r**, (0.15, 0.24998, 0.00001, 0.00001, 0.6) for panels **m** and **s**, (0.2, 0.24998, 0.00001, 0.00001, 0.55) for panels **n** and **t**, (0.25, 0.24998, 0.00001, 0.00001, 0.5) for panels **o** and **u**, or (0.3, 0.24998, 0.00001, 0.00001, 0.45) for panels **p** and **v**.

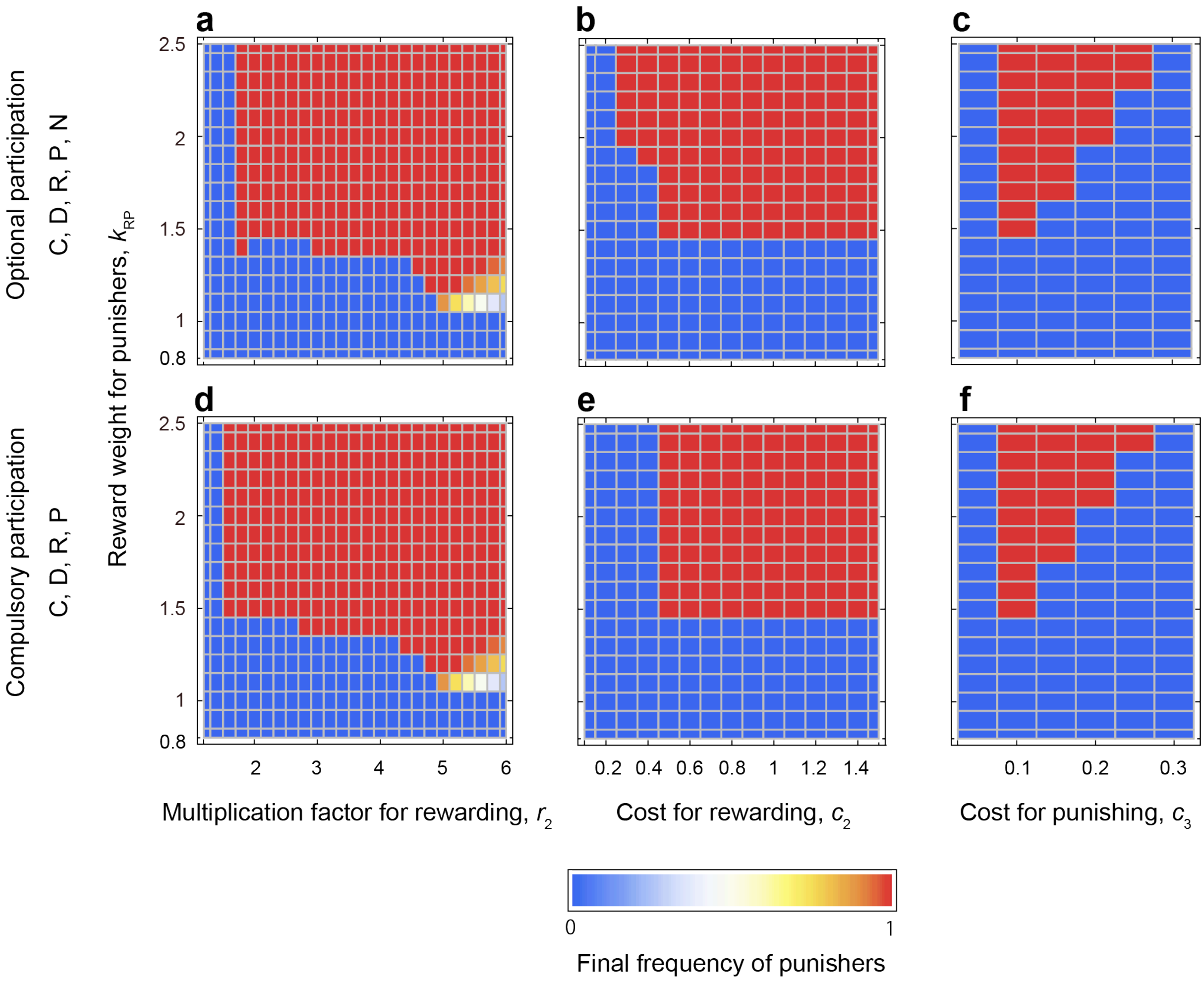

Figure S6 | Parameter analyses for different incentive multipliers, costs, and weights. Initial conditions are: (x_C, x_D, x_P, x_R, x_N) = (0.33, 0.338, 0.001, 0.001, 0.33) for panel **a**, (0.4, 0.298, 0.001, 0.001, 0.3) panels **b** and **c**, (0.33, 0.339, 0.33, 0.001, 0) for panel **d**, (0.4, 0.299, 0.001, 0.3, 0) for panels **e** and **f**. Other parameter values are as in Fig. 1.

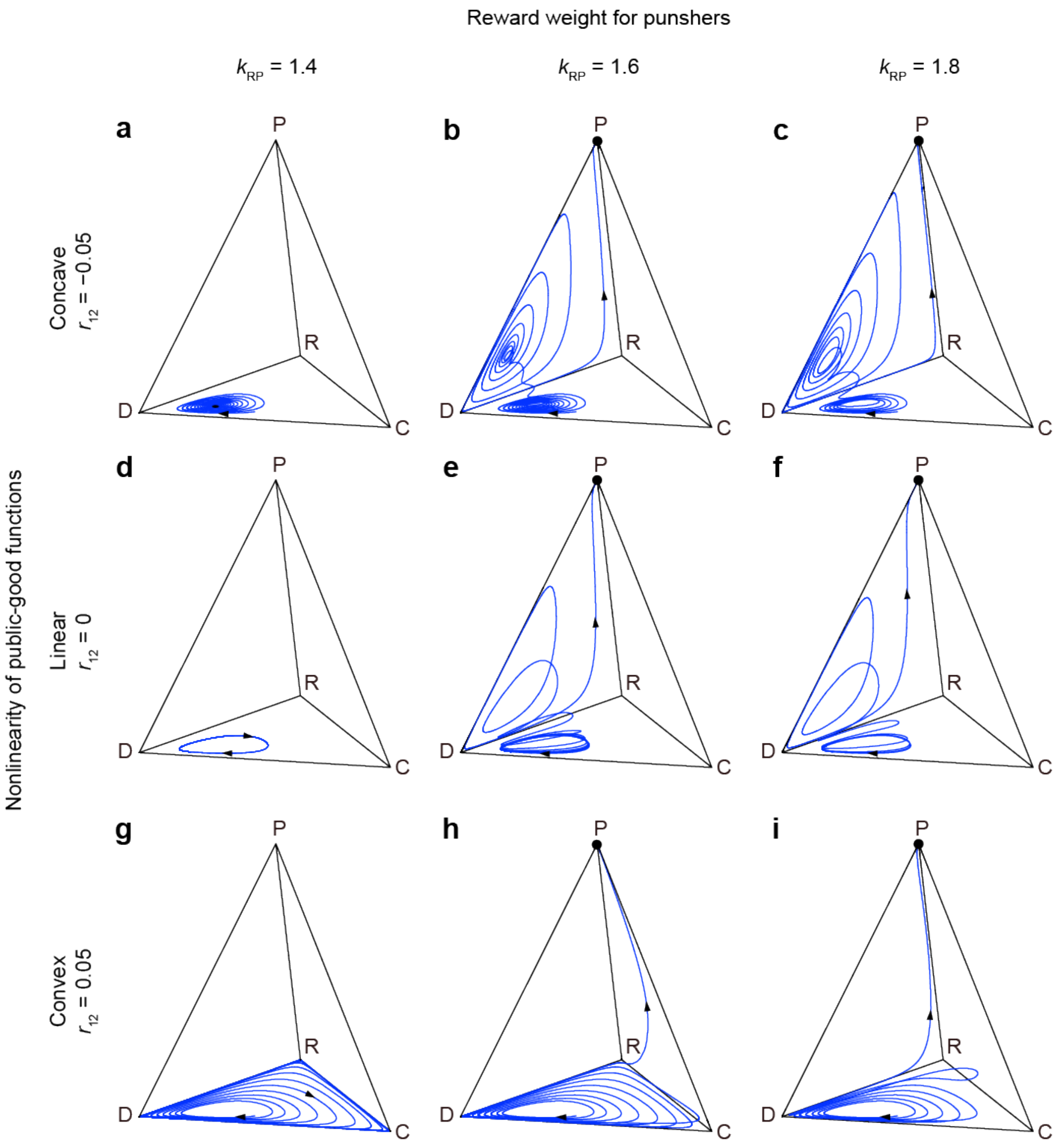

Figure S7-1 | Responses to nonlinearity of benefit functions in compulsory public good games with pool reward and punishment. The corresponding time series of the relative frequencies of four strategies, C, D, R, and P, are in Supplementary Fig. S7-2. (**a-c**) Concave benefit functions lead to an attractor on the CDR face. (**d-f**) Linear benefit functions lead to periodic closed orbits on the CDR face. (**g-i**) Convex benefit functions lead to a repeller on the CDR face. In spite of those differences in nonlinearity, the population state can eventually converge to the all-P state, as the reward weight for punishers k_{RP} are sufficiently large. Parameter values are as in Fig. 1. Initial conditions are: $(x_C, x_D, x_R, x_P) = (0.4, 0.49999, 0.1, 0.00001)$.

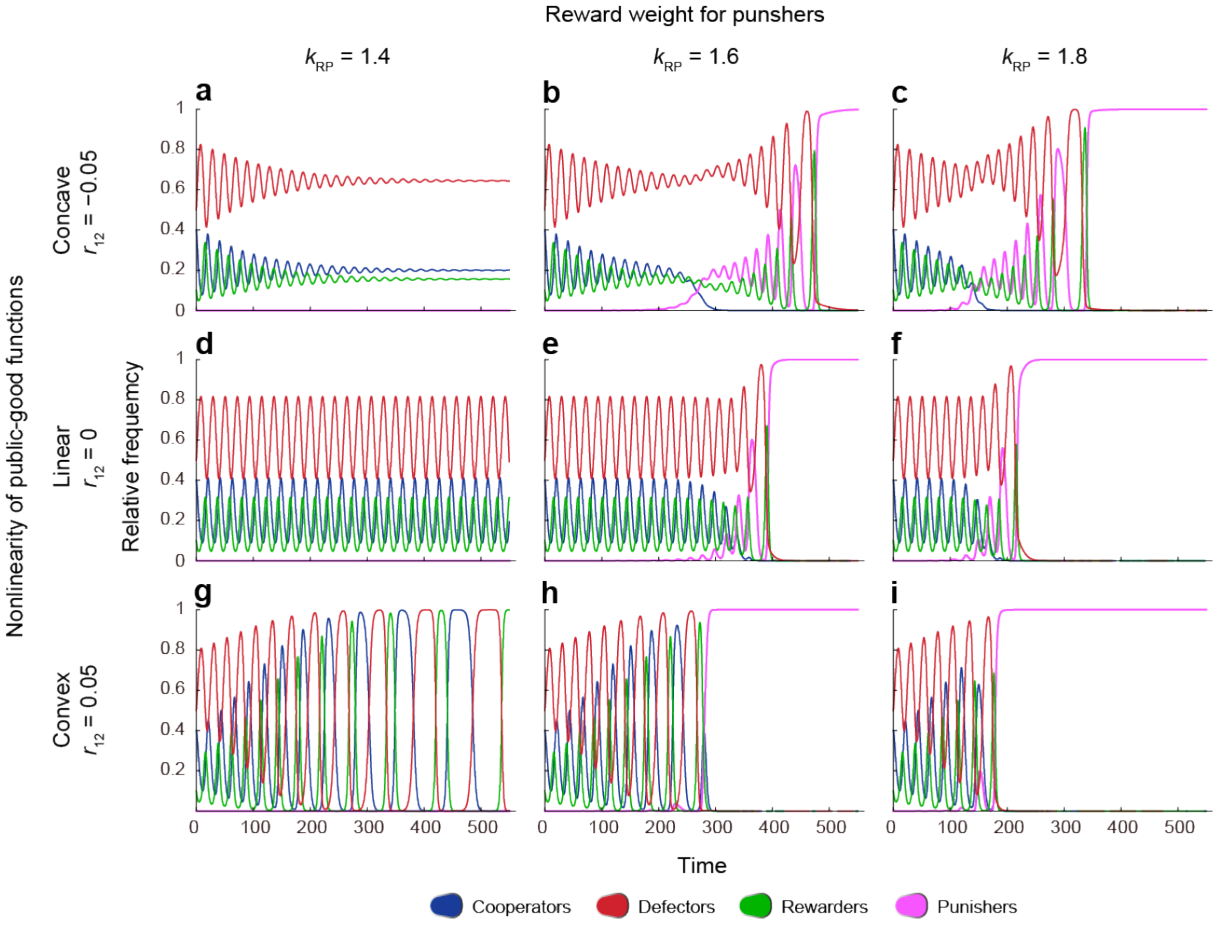

Figure S7-2 | Responses to nonlinearity of benefit functions in compulsory public good games with pool reward and punishment. Time series of the frequencies of four strategies, C (*blue*), D (*red*), R (*green*), and P (*purple*), corresponding to Supplementary Fig. S7-1.
